# ﻿Rediscovery of *Rubuspendulus* Rusby (Rosaceae) and a new record for the flora of Ecuador and Peru

**DOI:** 10.3897/phytokeys.227.100859

**Published:** 2023-06-02

**Authors:** David A. Espinel-Ortiz, Carla J. Rodríguez, Katya Romoleroux

**Affiliations:** 1 Laboratorio de Botánica Sistemática, Herbario QCA, Facultad de Ciencias Exactas y Naturales, Pontificia Universidad Católica del Ecuador, Av. 12 de Octubre 1076 y Vicente Ramón Roca, 170525 Quito, Ecuador Pontificia Universidad Católica del Ecuador Qutio Ecuador; 2 Herbario Padre Luis Sodiro (QPLS), Centro Cultural Biblioteca Ecuatoriana Aurelio Espinosa Pólit, José Nogales 69-22 y Francisco Arcos, 170301 Quito, Ecuador Herbario Padre Luis Sodiro (QPLS), Centro Cultural Biblioteca Ecuatoriana Aurelio Espinosa Pólit Qutio Ecuador

**Keywords:** Andes, Ecuadorian, Rubeae, taxonomy

## Abstract

We report the rediscovery of *Rubuspendulus* Rusby, “Mora India”, described in 1933 from Colombia and not mentioned again until the present study. We also update its distribution with eight new localities in Colombia, seven in Ecuador and one in Peru, being a new record for the flora of the latter two countries. This is the first time that *R.pendulus*’ stipules and flowers are found and detailed through a botanical description, illustrations and photographs. *Rubuspendulus* is morphologically differentiated from *R.bogotensis* Benth., *R.mollifrons* Focke, *R.porphyromallos* Focke and *R.urticifolius* Poir., with whom it was previously confused and we give a brief explanation on the type specimen status of *R.mollifrons* and *R.porphyromallos*.

## ﻿Introduction

*Rubus* L. presents ca 836 species classified in 10 subgenera; thus, being one of the most diverse genera of the Rosaceae family (Huang et al. 2023). Despite the cosmopolitan distribution of the genus, it is more abundant in Asia, where at least 208 species (139 endemic) were reported in China alone (Lingdi and Boufford 2003). South America contains a low *Rubus* diversity with fewer than 60 species classified in the native subgenus Rubus L., and the introduced subgenera *Batothamnus* (Focke) E.H.L.Krause and *Idaeobatus* Focke (Macbride 1938; Romoleroux 1996; Forzza et al. 2010; Romoleroux et al. 2014; Bernal et al. 2020; Espinel-Ortiz and Romoleroux 2020, 2021; Moreno-Medina et al. 2020; Huang et al. 2023). Thus, more focalized taxonomic studies will help to uncover more species of *Rubus* in the Neotropics.

The “Mora India”, *Rubuspendulus* Rusby (subgenus Rubus), is among the few *Rubus*’ vine species in South America. Its holotype is the only collection, and thus, the only locality known for this species, and its flowers’ description is non-existent (Rusby 1933). Despite the type locality in Colombia, this name was excluded from the Colombian Plant Catalogue (Bernal et al. 2020), and has not been mentioned for over 90 years. Curiously, this species is not the only one that should be revised for the Colombian flora. *Rubusmollifrons* Focke and *R.porphyromallos* Focke also should be reviewed, as both do not have any type collection. In fact, during their original description Focke (1911b) did not cite any sample, but affirmed that *R.mollifrons* is found in Colombia and *R.porphyromallos* could inhabit the South American Andes.

After careful examination of more than 3000 *Rubus* samples from different herbaria, specimens representing this species showed only a few samples and were often annotated as *R.bogotensis* Benth. *R.mollifrons*, *R.porphyromallos*, *R.urticifolius* Poir. or were unidentified. However, *R.pendulus* vegetative and reproductive characters differ greatly from those of the species reported for Colombia, Ecuador and Peru (Macbride 1938; Romoleroux 1996; Bernal et al. 2020; Moreno-Medina et al. 2020; Espinel-Ortiz and Romoleroux 2020, 2021). Here, we provide information to support *Rubuspendulus* as a valid species, its differences from other *Rubus* species and a brief explanation of the type status of *R.mollifrons* and *R.porphyromallos*. We also updated the key for the Ecuadorian *Rubus* species.

## ﻿Methodology

The *Rubus* collections of the Herbaria HA, HUTI, LOJA, NY, Q, QAP, QCA, QCNE, QPLS and QUSF were revised, and samples not fitting with the species reported for Ecuador were studied. Additional samples from AAU, COL, F, MO and US were revised from online images to cover the original distribution of this species in Colombia and see if it reached Peru. In total, ca 2500 samples of Ecuador, ca 700 samples of Colombia and one of Peru were revised. During 2021, we recollected more material near Quito (Ecuador) in several field trips in order to complete the species descriptions of its flowers and update the ecological data. A taxonomic key for the Ecuadorian *Rubus*’ species is provided as supplementary material (See Suppl. material 1).

To categorize *R.pendulus* as a valid species, we used the *Rubus* species definition proposed by Weber (1977) and Haveman and Ronde (2013). These proposals suggest that a widely-distributed biotype, whose diameter of distribution area goes from 500 km to more than 1000 km, can be considered a species.

The botanical terms used in the descriptions followed those used by Stearn (1986), and the pubescence types were based on the terminology of Hickey and King (2000), and Wilhelm and Rericha (2020). Some specimens examined for the description (e.g. D. Espinel-Ortiz & H.G. Abad 300) were mounted in more than one herbarium sheet, and/or have additional dry or alcohol material; therefore, each part had its own herbarium barcode (QAP and QCA). For these samples, we wrote all the herbarium barcodes for each part in examined specimens when available. We used QGIS (QGIS.org 2022) for the distribution map, using the geographic coordinates from the samples. Additionally, geographic reference coordinates based on locality description were selected for the samples from Colombia that lacked this information.

## ﻿Taxonomic treatment

### ﻿ Rubus pendulus

Taxon classificationPlantaeRosalesRosaceae

Rusby, Torreya 33:41. 1933.

DB2EC27D-7B1F-5F04-AC51-B265125BBCC2

Figs 1 , 2 , 3

#### Type.

**Colombia. Huila**: Balsillas, at Balsillas river, edge of forest, 2000–2100 m, 03–05 Aug 1917, *H.H. Rusby & F.W. Pennell 719* (holotype: NY (NY-424649)).

**Figure 1.**
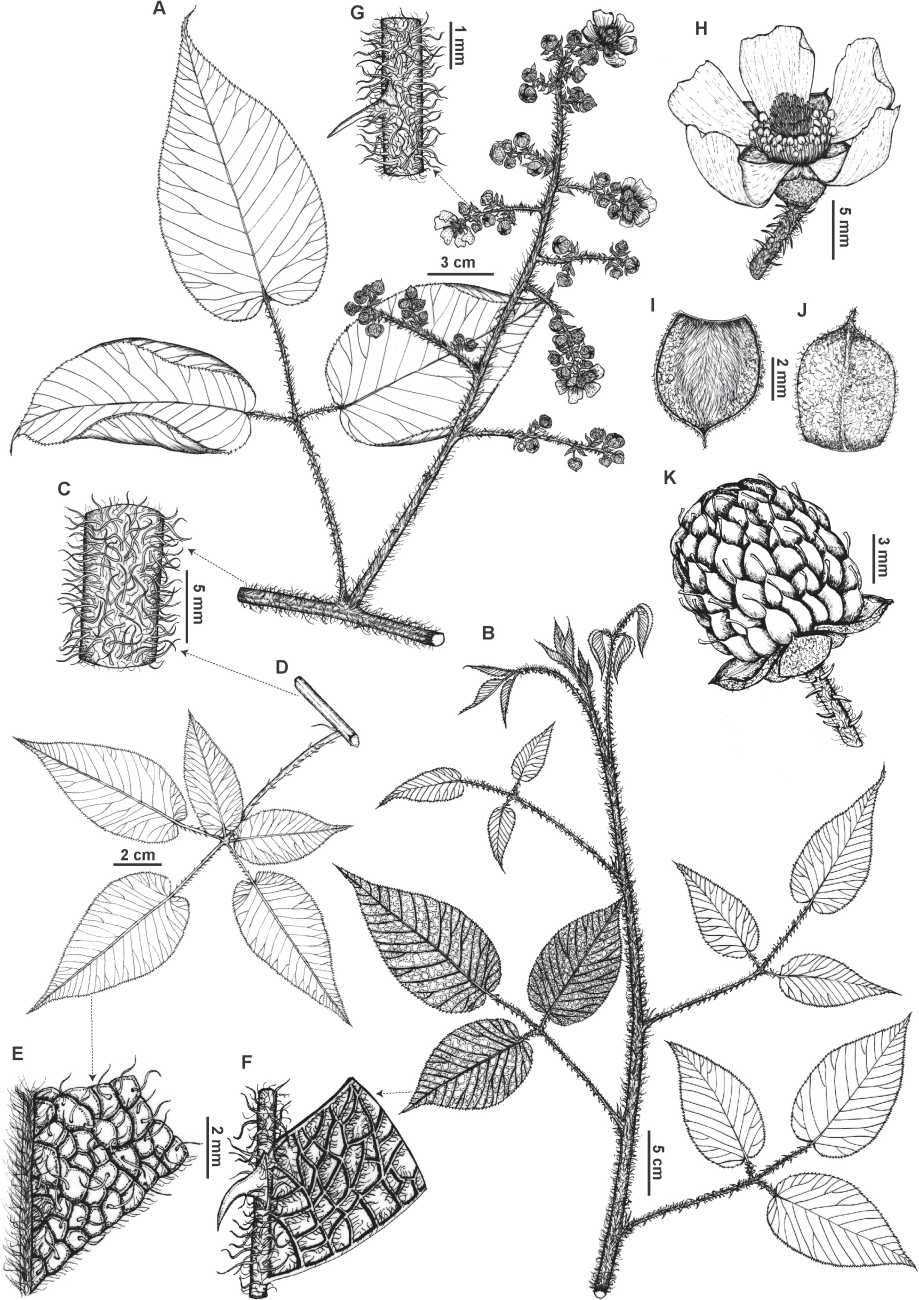
*Rubuspendulus* Rusby **A** inflorescence **B** habit and leaves **C** branch **D** 5-foliolate leaf **E** leaf adaxial surface **F** leaf abaxial surface **G** pedicel **H** flower **I** sepal adaxial surface **J** sepal abaxial surface **K** fruit (**K** based on Fernández et al. 606 (QCNE) **B** based on Espinel-Ortiz et al. 301 (QCA) **C–F** based on Espinel-Ortiz et al. 304 (QCA), **A, G–J** based on Espinel-Ortiz et al. 382 (QCA)). Illustrations by Carla Rodríguez.

#### Description.

**Woody vine** growing up to 10 m long, **or scandent or climbing shrub**, with all prickles from the base 1⁄3–2⁄3 sparsely villous-hirsute with red setose hairs, glabrous towards the apex, eglandular or with subsessile glands. **Branches** obtuse-angled, red to slightly brownish, with red setose hairs, and hirsute, 3.4–9.4 mm diam., eglandular or with some setose hairs ending in a gland, unarmed or with up to 5 prickles (per total area of 5 cm long of the branch), gradually narrowed from a broad base, curved at the apex, 1.1–4.1 × 1.2–7.4 mm. **Stipules** asymmetrically narrow, subulate, 4.7–9.7 × 0.4–1.8 mm, margin entire, chartaceous; adaxial surface sparsely hirsute on veins, with red subsessile glands on margin; abaxial surface with red setose hairs on veins and towards the margin, and hirsute, with red sessile and subsessile glands. **Petioles** 4.8–10.6 (–15.2) cm long, with red setose hairs, and hirsute, with 17–35 prickles, gradually narrowed from a broad base, curved at the apex, 0.9–2.8 × 0.6–2.8 mm; basal petiolules 3.0–6.5 mm long, unarmed or with up to 5 prickles, gradually narrowed from a broad base, curved at the apex, 0.7–1.1 × 0.3–1.1 mm; lateral petiolules 7.0–29.7 (–45.5) mm long, with 3–17 (–34) prickles, gradually narrowed from a broad base, curved at the apex, 0.6–2.0 × 0.4–1.6 mm; terminal petiolules 2.8–5.1 (–9.0) cm long, with 8–27 (–42) prickles, gradually narrowed from a broad base, curved at the apex, 0.6–2.8 × 0.4–3.1 mm. **Leaves** trifoliate to 5-foliate; leaflets ovate to elliptic, base subcordate or asymmetrically subcordate, apex acuminate, margin serrate or bidentate towards the apex, basal leaflets (2.6–) 5.3–7.7 × (1.1–) 2.5–4.0 cm, lateral leaflets 7.1–14.3 × 3.7–7.0 cm, terminal leaflet (6.7–) 10.4–15.1 × (3.8–) 4.8–7.3 cm, chartaceous, with 10–19 secondary veins, adaxial surface bullate, sparsely villous-hirsute on each bubble, and densely villous hirsute on the midvein and secondary veins, with red subsessile glands, unarmed, abaxial surface glabrous with red setose hairs, and villous only on the veins, with red subsessile glands on the veins, rarely unarmed or with 8–18 (32) prickles on the primary vein, gradually narrowed from a broad base, curved at the apex, 0.2–1.8 × 0.3–2.1 mm. **Inflorescences** compact, compound, terminal and axillary cymes, 6–53-flowered, 8.2–15.3 cm long, with simple or trifoliate leaves below; peduncles terete, red to slightly brownish, (5.7–) 8.1–20.3 (–48.7) mm long, villous with abundant red setose hairs, eglandular, unarmed or with up to 14 prickles, gradually narrowed from a broad base, curved at the apex, 1.4–2.2 × 0.4–1.6 mm; pedicels terete, red to slightly brownish, 2.4–7.5 (–9.1) mm long, villous with abundant red setose hairs, eglandular, with 6–20 prickles, gradually narrowed from a broad base or triangular, curved at the apex, 0.8–2.4 × 0.1–1.4 mm. **Flowers** 8.21–15.25 mm diam.; sepals 5, obovate to elliptic or slightly lanceolate, apex mucronulate, margin entire, 5.1–7.9 × 2.5–4.6 mm, greenish-red to red, adaxial surface concave, villous-sericeous, and tomentose towards the apex and the margin, with sessile and subsessile glands, unarmed, abaxial surface convex, tomentose, with subsessile glandular, unarmed; petals 5, narrowly obovate to elliptic, margin entire, 9.0–13.9 × 6.1–7.8 mm, fuchsia to pink, glabrous, eglandular, adaxial surface deeply concave, abaxial surface deeply convex, stamens with anthers glabrous, filaments pale pink, glabrous; pistils, stigmas glabrous, styles slightly hirtellous, ovaries densely villous. **Fruits** green to red when immature, and black at maturity, ovoid to globose, 7.8–15.4 × 6.6–11.0 mm (when dry); drupelets 66–115 per receptacle, 2.1–4.3 × 1.1–2.8 mm (when dry), sparsely villous.

**Figure 2.**
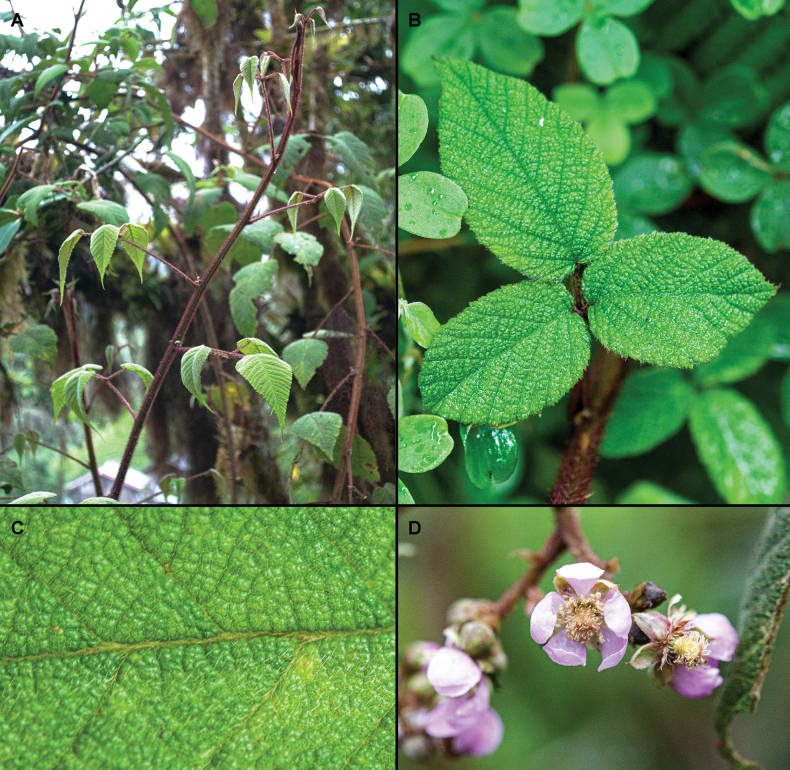
*Rubuspendulus* Rusby **A** habit **B** trifoliate leaf adaxial surface **C** bullate leaf adaxial surface **D** flowers. Photos by David A. Espinel-Ortiz.

#### Specimens examined.

**Colombia. — Huila**: Neiva, Vereda La Plata, Finca La Colonia (Antigua Carolina), 2000 m, 31 Oct 1996 (fl), *F. Llanos & W.F. Gerardino 2797* (COL). — **Magdalena**: Sierra Nevada de Santa Marta, Finca Cecilia, Quebrada Indiana, ca 10°59.000'N, 73°58.000'W, ca 1750 m, 03 Sep 1972 (fl), *J.H. Kirkbride 2082* (COL); Sierra Nevada de Santa Marta, Finca Los Arroyitos, ca 10°56.000'N, 73°58.000'W, ca 1800 m, 07 Oct 1972 (fl, fr), *J.H. Kirkbride 2436* (COL, US (US-3733777)). — **Santander**: Between Piedecuesta and Las Vegas, 2000–2500 m, 19–24 Dec 1926 (fr), *E.P. Killip & A.C. Smith 15567* (NY); Municipio Onzaga, Vereda Chaguacá, 2640 m, 30 Mar 1976 (fr), *J.H. Torres, G. Lozano & S. Díaz 539* (COL). — **Cundinamarca**: Facatativá, Alto de Peña Negra, 2810–2820 m, 29 May 1941 (fl, fr), *H. García-Barriga & R. Jaramillo 104033* (US (US-3733540)). — **Bogotá-DC**: 25 miles SW of Bogotá, 18 Mar 1952 (fr), *G.M. Darrow s.n.* (US (US-3733541)). — **Cesar**: Municipio Valledupar, Corregimiento de Puerto Bello, 1200–2000 m, 13 Jul 1983 (fl), *Cuadros H.V. 1685* (COL). **Ecuador. — Pichincha**: San José de Mindo, Nono-Tandayapa road, route of the OCP Heavy Crude Oil Pipeline, Cerro Castillo and La Bola, 00°01.750'S, 78°40.984'W, 2600 m, 05 Oct 2001 (fl, fr), *D. Fernández, E. Toapanta, M. Mites & C. Morales 606* (MO, QCNE (QCNE-159936)); Quito, Nanegalito, vía a San Tadeo, Área Protegida Privada Bellavista, 00°02.170'S, 78°42.067'W, 2297 m, 03 Dec 2021, *D.A Espinel-Ortiz & H.G. Abad 300* (QCA (QCA-244065, QCA-7010819 to QCA-7010822 and QCA-7010828)); same locality as for preceding, 00°02.178'N, 78°42.227'W, 2297 m, 03 Dec 2021, *D.A Espinel-Ortiz & H.G. Abad 301* (QCA (QCA-244068 and QCA-7010829 to QCA-7010831)); Quito, Nanegalito, vía al Área Protegida Privada Bellavista desde carretera E26, 00°00.077'N, 78°41.356'W, 2281 m, 07 Dec 2021, *D.A Espinel-Ortiz & H.G. Abad 303* (QCA (QCA-244067 and QCA-7010825 to QCA-7010827)); same locality as for preceding, 00°02.178'S, 78°42.227'W, 2315 m, 20 Apr 2022, *D.A Espinel-Ortiz & H.G. Abad 327* (QCA); same locality as for preceding, 00°02.274'S, 78°42.275'W, 2303 m, 20 Apr 2022, *D.A Espinel-Ortiz & H.G. Abad 328* (QCA); same locality as for preceding, 00°02.281'S, 78°42.316'W, 2 m, 16 May 2022 (fl), *D.A Espinel-Ortiz & H.G. Abad 382* (QCA); Quito, Nanegalito, El Golán, between El Alí and El Porvenir, 00°06.570'N, 78°35.150'W, 2444 m, 25 May 2021 (fl), *C.E. Cerón & C.I. Reyes-Tello 88459* (QAP (QAP-106468 and QAP-106757)); Quito, Nanegalito, El Golán, between Edén Mágico and El Porvenir, 00°05.270'N, 78°33.230'W, 2402 m, 10 Jul 2021, *C.E. Cerón, C.I. Reyes-Tello, D. Bacuilima & A. Acosta* 88667 (QAP (QAP-106886)); Quito, Yunguilla, pasando la entrada a la comunidad El Golán, 00°06.485'N, 78°33.207'W, 2641 m, 08 Dec 2021, *D. Espinel-Ortiz & H.G. Abad* 304 (QCA (QCA-244066 and QCA-7010824). — **Napo**: National Park Los Llanganates, Salcedo-Tena road, km 60, “La Poderosa’’ ranch, descending to Mulatos river, 4 km, 00°57.000'S, 78°14.000'W, 2500–2870 m, 16 Mar 1995 (fl, fr), *H. Vargas & D. Sandoval 451* (MO (MO-1610744), NY). — **Loja**: Ca. 5 km of Paso de Sabanilla, on road Yangana-Valladolid, 04°27.00'S, 79°10.000'W, 2500 m, 03 Sep 1985, *S. Lægaard 55178* (AAU). — **Morona Santiago**: Sangay National Park, Guamote-Macas road, near Purshi-Zuña, 02°11.000'S, 78°20.000'W, 2400–2700 m, 07 Jun 1998, *C.E. Cerón 36281* (QAP (QAP-91)). — **Zamora Chinchipe**: Nanguipa Cordillera, Cerro Colorado, about 8 km by air SSE of Nambija, 20 km ESE of Zamora, montane cloud forest, 04°07.483'S, 78°46.417'W, 2500 m, 18 Feb 2002 (fr), *D. Neill, W. Quizhpe, J. Manzanares, A. Hirtz, T. DeLinks & C. Cole 13778* (MO, QCNE (QCNE-162651)); Parque Nacional Yacuri, San Andrés, colecciones en la vía Jimbura-Zumba, ca 500 m del río Isimanchi, 04°47.100'S, 79°22.668'W, 2653 m, 29 Apr 2015, *Á.J. Pérez, N. Zapata, W. Santillán & R. Jiménez 8997* (QCA (QCA-233885)). **Peru. — Cajamarca**: Cutervo, San Andrés de Cutervo, Parque Nacional Cutervo, arriba de Sucedal pasando por Chorro Blanco, 06°11.353'S, 78°41.578'W, 2250 m, 03 Aug 1988 (fr), *C. Díaz & H. Osores 2942* (F, MO).

**Figure 3.**
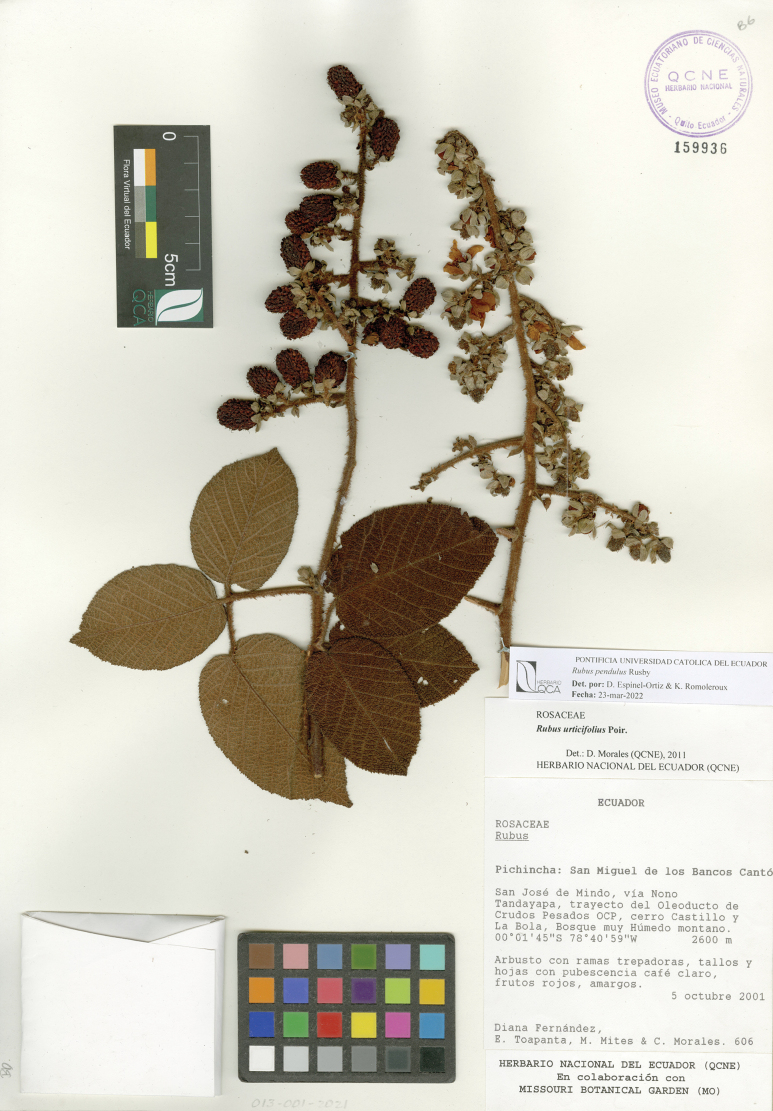
*Rubuspendulus* Rusby. Collection Fernández et al. 606 (QCNE) with flowers and fruits.

#### Distribution.

**Figure 4.**
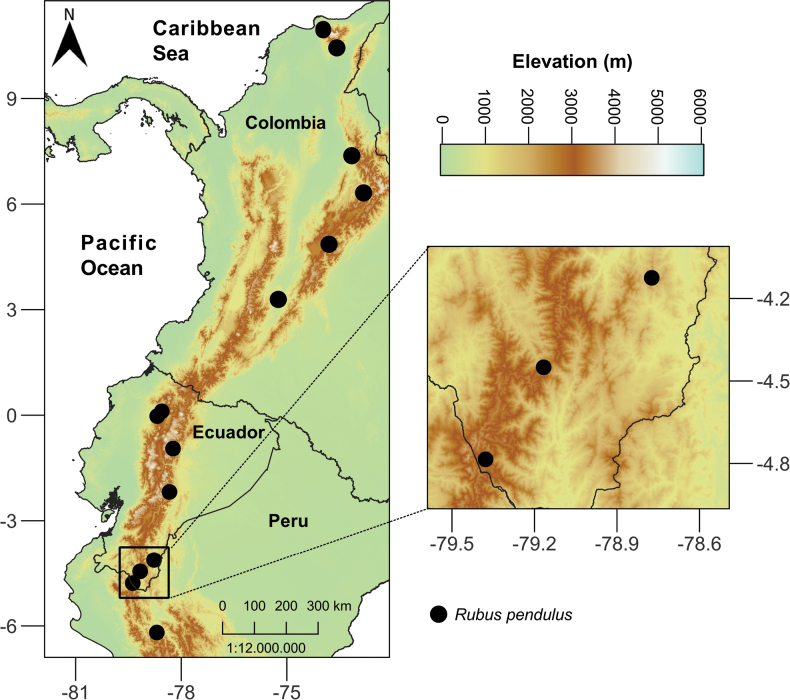
Distribution map of *Rubuspendulus* (black circle) in Colombia, Ecuador and Peru.

*Rubuspendulus* is distributed in the Northern and Central Andes (Fig. 4). In Colombia, it is known from seven collections in Bogotá DC and the Departments of Cesar, Cundinamarca, Huila, Magdalena and Santander. In Ecuador it was found in the provinces of Loja, Morona Santiago, Napo, Pichincha and Zamora Chinchipe. Lastly, from Peru it is known from one collection in Cajamarca. This species inhabits the Andean cordillera from 2000 to 2900 m a.s.l.; however, there were two specimens from Magdalena which showed a lower distribution from ca 1700 to 1800 m.

#### Ecology.

This species occurs in montane cloud forests dominated by trees and shrubs and in nearby disturbed areas. *Rubuspendulus* can be found living in sympatry with *Rubusadenotrichos* Schltdl., *R.boliviensis* Focke, *R.longistipularis*, *R.porphyromallos* and *R.urticifolius*. Flowering and fruiting collections dated from February, March, May and October.

#### Conservation status.

*Rubuspendulus* is known from at least 18 localities, impacted by human activities, including regression to agriculture and road openings. Following the IUCN (2022) guidelines, based on the geographic distribution and altered land use at the localities, this species should be categorized as least concern (LC).

## ﻿Discussion

*Rubuspendulus* was described by Rusby (1933) with a sample collected at Balsilla’s river, Balsillas, Huila Department in Colombia. All the revised material agrees with the original description and resembles the holotype collection. The most conspicuous characteristics from both, the holotype and the material examined here, are the setose hairs and hirsute pubescence referred in by Rusby as ferrugineous-tomentose, long petioles (4.8–10.6 cm long), bullate leaves, long and slightly thin leafletes (5.3–15.1 × 2.5–7.3 cm) with subcordate base, the secondary veins pattern (at an angle of about 45 degrees with the main vein), concave sepals with a mucronulate apex referred by Rusby as apiculate, and compact (crowded) and nearly subsessile fruits with small drupelets (2.1–4.3 × 1.1–2.8 mm).

Since its description (Rusby 1933), no other records of *R.pendulus* were reported until Romoleroux (1996) suggested that sample Lægaard 55178 (AAU) may belong to this species. However, the available material could not be properly identified as it lacked flowers and fruits. During this revision, several samples annotate here as *R.pendulus*, were previously identified as *R.bogotensis*, *R.mollifrons*, *R.porphyromallos* or *R.urticifolius*. From these, only the latter two show similar characteristics to *R.pendulus*, whereas *R.bogotensis* and *R.mollifrons* have almost nothing in common with it.

*Rubusbogotensis* is characterized for is abundant shortly stipitate glands covering all the plant, absence of setose hairs, long pedicels (5–20 mm long) and big fruits (15–20 × 10–20 mm) with only a few drupelets (10–35) per fruit (Romoleroux, 1996). On the other hand, *R.pendulus* has red setose hairs covering all the plant, glands only in some of the setose hairs, shorter pedicels (2.4–7.5 mm long) and smaller fruits (7.8–15.4 × 6.6–11 mm) with more drupelets (66–115) per fruit. In addition, *R.pendulus*’ bullate leaves differentiate it from *R.bogotensis* and the other species, as this is only a characteristic previously found in *R.azuayensis* Romol. and *R.betonicifolius* Focke, both simple-leaf species (See Suppl. material 2).

*Rubuspendulus* may resemble *R.urticifolius* by its red setose hairs, mostly eglandular trifoliate to 5-foliate leaves, and ovate to elliptic leaflets, but it differs from the latter by its bullate leaves, few flowered inflorescences (up to 60 flowers), and mucronulate sepals in contrast with the non-bullate leaves, many flowered inflorescences (60–150 flowers), and apiculate or acuminate sepals of *R.urticifolius*. Furthermore, *R.pendulus* has bigger fruits (7.8–15.4 × 6.6–11 mm) with more (66–115) and bigger drupelets (2.1–4.3 × 1.1–2.8 mm), whereas *R.urticifolius* has smaller fruits (7–10 × 6–9 mm) with fewer (30–50) and smaller drupelets (1.5–3 × 1–2 mm) (See Suppl. material 2).

The two species mentioned before were registered in Colombia, Ecuador, Peru and Bolivia (Romoleroux 1996). However, *R.mollifrons* was recorded only from Colombia and *R.porphyromallos* was said to inhabit the South American Andes (Focke 1911a, 1911b). These two species lack a holotype as Focke did not mention any sample during their original description (Focke 1911b). Even in his monograph (Focke 1911a), no sample was cited for either species. Following the Shenzen Code’s art. 9, as no sample or illustration was presented in the protologue, neotypes should be selected for both species (Turland et al. 2018). Luckily, Focke (1911a) included a photograph of *Rubusmollifrons* collection in his monograph; should this sample be found, it could be designated as the neotype for this species. On the other hand, no illustration of *R.porphyromallos* was included; therefore, a neotype following the species description should be selected. However, for both cases, before the designation of any neotype, an extensive revision of historic Colombian *Rubus* samples is necessary. For this reason, here, we proceed to differentiate *R.pendulus* from both species based on their descriptions (Focke 1911a, 1911b). Also, we give a brief explanation of the identity of the samples identified as *R.porphyromallos* in COL and NY.

*Rubusmollifrons* is described as a climbing shrub with tomentose stems; short, tomentose petioles, lateral petioles ca 1 cm long, and terminal petiolule ca 2 cm long; linear-lanceolate stipules; trifoliate leaves, with leaflets oblong-ovate, base subcordate, apex acuminate, 6–8 × 4–5 cm and 10–12 secondary veins; leaf adaxial surface densely pubescent and abaxially grayish-pannose (“canescent-velutina”); the inflorescences are grayish-tomentose, subarmed, pauciflora or uniflora; the flowers are short-peduncled, ca 5 cm; sepals ovate and grayish-tomentose; petals elliptic, white or slightly pink on the outside, and the petals are shorter than the sepals; no fruits observed (Focke 1911a, 1911b).

Focke (1911a, 1911b) described *R.porphyromallos* as a shrub covered in two kinds of pubescences: the first one is said to be “rufous-villous” or reddish-villous, and the other one tomentose. The stems are eglandular, with prickles; long petioles with prickles, basal petiolules 2–2.5 cm long, lateral petiolules ca 4 cm long, terminal petiolules 5–6 cm long; palmate-compound 5-foliolate leaves, leaflets oblong-ovate or ovate, base emarginate or subcordate, apex pointed, margin unevenly serrated, 15 × 10 cm, leathery, with 12–15 secondary veins; adaxial surface strigose; abaxial surface softly grayish-pannose (“canescenti-velutina”), young leaflets white; broad compound inflorescences, tomentose-villous, with prickles and trifoliate leaves; flowers shortly pedicellate, ca 1.5 cm diam.; sepals ovate, apex acute or minute, greyish-tomentose, not villous; petals obovate; stamens shorter than sepals; no fruits observed.

In Focke’s original description, the latin word “velutina” is literally translated to velvet; the equivalent pubescence is pannose, as he used the same word to describe the pubescence of *R.boliviensis* holotype which is pannose (Romoleroux, 1996). It is also worth mentioning that Focke already used the term bullate in *R.betonicifolius*, and red-setose pubescence as “rufo-setosi” in *R.urticifolius* (annotated as “*R.urticaefolius*”) (Focke 1911a), as both are among the most conspicuous characteristics of *R.pendulus* and were not mentioned for either *R.mollifrons* or *R.porphyromallos* (Focke 1911a, 1911b). *Rubuspendulus* differs from *R.mollifrons* by having red setose hairs all over the plant, longer lateral (0.7–2.97 cm long) and terminal (2.8–5.1 cm long) petiolules, bullate leaves, ovate to elliptic and bigger (5.3–15.1 × 2.5–7.3 cm) leaflets, with more secondary veins (10–19), leaf abaxial surface glabrous with red setose hairs, and villous only on the veins, more flowers (6–56) per inflorescence, shorter peduncles (0.8–2.0 mm long), sericeous-villous and tomentose sepals (See Suppl. material 2).

In the case of *R.porphyromallos*, its description has similar characteristics to that of *R.pendulus*, but it presents some differences such as eglandular stems, longer basal petiolules (2–2.5 cm long), non-bullate leaves, broader leaflets (15 × 10 cm), leaf abaxial surface pannose, ovate and greyish-tomentose, not villous sepals. Whereas *Rubuspendulus* has some red setose hairs ending in glands, shorter basal petiolules (0.3–0.7 cm long), bullate leaves, thinner leaflets (5.3–15.1 × 2.5–7.3 cm), leaf abaxial surface glabrous with red setose hairs, and villous only on the veins, obovate to elliptic or slightly lanceolate, sericeous-villous and tomentose sepals (See Suppl. material 2). Another difference could be the absence of setose hairs in *R.porphyromallos* as this term was not mentioned in its original description (Focke 1911b).

### ﻿Why has the name *Rubusporphyromallos* been widely used in Colombia?

Most of the Colombian collections identified here as *R.pendulus* were collected between the 1970s and the 2000s. However, sample Killip & Smith 15567 was collected in 1926 and identified by Killip as *R.porphyromallos* in 1932. Interestingly, Rusby described *R.pendulus* the next year, but he never saw the collection from Killip. The same way, Killip never saw Rusby’s collection as both worked in different herbaria, with most of Killip’s samples deposited in US, and Rusby’s in NY. Taking into account that Focke (1910, 1911a, 1914) did not cite many samples of Colombia, it is possible that because of Killip’s ongoing field trips and extensive work and influence in the Colombian flora, his *Rubus* identifications were used as a reference to identify this genus. So it is that the name *R.porphyromallos* has been conserved for samples that were highly similar until recent years (COL, NY).

## ﻿Conclusions

*Rubuspendulus* is a widely spread species from the north of South America that has been poorly collected before and thus confused with different species. However, morphologically it is different from other similar species. More collection efforts are necessary to have an assessment of this species’ complete distribution. Additionally, as *R.porphyromallos* showed the closest resemblance to *R.pendulus*, it is fundamental to designate a neotype for *R.porphyromallos* and study both of them genetically to understand their evolutive history.

## Supplementary Material

XML Treatment for Rubus pendulus

## ﻿Additional information

### Author ORCIDs

David A. Espinel-Ortiz https://orcid.org/0000-0003-3405-0375

Carla J. Rodríguez https://orcid.org/0000-0002-4204-1316

Katya Romoleroux https://orcid.org/0000-0002-0679-9218

### Data availability

All of the data that support the findings of this study are available in the main text or Supplementary Information.

## Supplementary materials

Supplementary material 1 Taxonomic identificacion key for Ecuadorian *Rubus*’ species Data type Taxonomic identificacion key (table) https://binary.pensoft.net/file/858867This dataset is made available under the Open Database License (http://opendatacommons.org/licenses/odbl/1.0/). The Open Database License (ODbL) is a license agreement intended to allow users to freely share, modify, and use this Dataset while maintaining this same freedom for others, provided that the original source and author(s) are credited.David A. Espinel-Ortiz, Carla J. Rodríguez, Katya Romoleroux

Supplementary material 2 Comparison of main morphological characters between *Rubuspendulus*, *R.urticifolius*, *R.porphyromallos*, *R.bogotensis* and *R.mollifrons* Data type Morpholical differences between five *Rubus* species (table) https://binary.pensoft.net/file/858868This dataset is made available under the Open Database License (http://opendatacommons.org/licenses/odbl/1.0/). The Open Database License (ODbL) is a license agreement intended to allow users to freely share, modify, and use this Dataset while maintaining this same freedom for others, provided that the original source and author(s) are credited.David A. Espinel-Ortiz, Carla J. Rodríguez, Katya Romoleroux

## References

[B1] Bernal R Gradstein SR Celis M [Eds] (2020) Catálogo de Plantas y Líquenes de Colombia. v1.1. Universidad Nacional de Colombia. Dataset/Checklist. 10.15472/7avdhn

[B2] Espinel-Ortiz DA Romoleroux K (2020) *Rubusrosifolius* Smith: A new record of an alien species in the flora of Ecuador. BioInvasions Records 9(4): 712–722. https://www.reabic.net/journals/bir/2020/4/BIR_2020_Espinel-Ortiz_Romoleroux.pdf

[B3] Espinel-Ortiz DA Romoleroux K (2021) Two new species of *Rubus* L. (Rosaceae) from the western Andes of Ecuador. PhytoKeys 187: 141–159. 10.3897/phytokeys.187.76963 35068972 PMC8712496

[B4] Focke WO (1910) Species Ruborum, Monographiae generis Rubi prodromus part I. Stuttgart, E. Schweizerbart, New York (NY), USA, 1–120. 10.5962/bhl.title.15533

[B5] Focke WO (1911a) Species Ruborum, Monographiae generis Rubi prodromus part II. Stuttgart, E. Schweizerbart, New York (NY), USA, 120–223. 10.5962/bhl.title.15533

[B6] Focke WO (1911b) Rubi novi Americae australis et centralis. I. Feddes Repertorium Specierum Novarum Regni Vegetabilis 9(13–15): 235–237. 10.1002/fedr.19110091311

[B7] Focke WO (1914) Species Ruborum, Monographiae generis Rubi prodromus part III. Stuttgart, E. Schweizerbart, New York (NY), USA, 224–498.

[B8] Forzza RC Leitman PM Costa A de Carvalho AA Peixoto AL Walter BMT Bicudo C Zappi D da Costa DP Lleras E Martinelli G de Lima HC Prado J Stehmann JR Baumgratz JFA Pirani JR Sylvestre L da S Maia LC Lohmann LG Paganucci L Silveira M Nadruz M Mamede MCH Bastos MNC Morim MP Barbosa MR Menezes M Hopkins M Secco R Cavalcanti T Souza VC (2010) Catálogo de Plantas e Fungos do Brasil, volume 2. Instituto de Pesquisas Jardim Botânico do Rio de Janeiro, Rio de Janeiro, Brasil 1544. 10.7476/9788560035090

[B9] Haveman R Ronde ID (2013) The role of the Weberian Reform in European *Rubus* research and the taxonomy of locally distributed species–which species should we describe? Nordic Journal of Botany 31(2): 145–150. 10.1111/j.1756-1051.2012.01558.x

[B10] Hickey M King C (2000) The Cambridge Illustrated Glossary of Botanical Terms. Cambridge University Press, United Kingdom, 1–208.

[B11] Huang TR Chen JH Hummer KE Alice LA Wang WH He Y Yu S-X Yang M-F Chai T-Y Zhu X-Y Ma L-Q Wang H (2023) Phylogeny of *Rubus* (Rosaceae): Integrating molecular and morphological evidence into an infrageneric revision. Taxon 72(2): 278–306. 10.1002/tax.12885

[B12] IUCN (2022) Guidelines for Using the IUCN Red List Categories and Criteria, Version 15.1 Prepared by the Standards and Petitions Committee. https://nc.iucnredlist.org/redlist/content/attachment_files/RedListGuidelines.pdf [Accessed 17.03.2023]

[B13] Lingdi L Boufford D (2003) 28. *Rubus* Linnaeus, Sp. P1. 1:492. 1753. In: Wu Z Raven P Hong D (Eds) Flora of China, vol.9. Science Press, Beijing, China; Missouri Botanical Garden Press, St. Louis, USA, 195–285.

[B14] Macbride JF (1938) Rosaceae. In: Flora of Peru. Field Mus. Nat. Hist. Botanica Serbica 13(2,3): 1063–1119.

[B15] Moreno-Medina BL Casierra-Posada F Albesiano S (2020) *Rubusalutaceus* (Rosaceae), a new species for Colombia with agronomic potential. Revista Brasileira de Fruticultura 42(2): e-542. 10.1590/0100-29452020542

[B16] QGIS.org (2022) QGIS Geographic Information System. QGIS Association. http://www.qgis.org

[B17] Romoleroux K (1996) Rosaceae. In: Harling G Andersson L (Eds) Flora of Ecuador, vol.56. University of Gothenburg, Göteborg, Sweden; Riksmuseum, Stockholm, Sweden; Pontificia Universidad Católica del Ecuador, Quito, Ecuador, 1–151.

[B18] Romoleroux K Meneses RI Achá S (2014) Rosaceae. In: Jørgensen PM Nee MH Beck SG Arrázola S Saldías M (Eds) Catálogo de las Plantas Vasculares de Bolivia, vol.2. Missouri Botanical Garden Press, St. Louis, USA, 1131–1140.

[B19] Rusby HH (1933) A new blackberry from Colombia. Torreya 33(2): 41–43.

[B20] Stearn WT (1986) Botanical Latin: History, Grammar, Syntax, Terminology and Vocabulary, third Edition. David & Charles Publishers plc, Great Britain, 1–555.

[B21] Turland NJ Wiersema JH Barrie FR Greuter W Hawksworth DL Herendeen PS Knapp S Kusber W-H Li D-Z Marhold K May TW McNeill J Monro AM Prado J Price MJ Smith GF [Eds] (2018) International Code of Nomenclature for algae, fungi, and plants (Shenzhen Code) adopted by the Nineteenth International Botanical Congress Shenzhen, China, July 2017. Regnum Vegetabile 159. Koeltz Botanical Books, Glashütten. 10.12705/Code.2018

[B22] Weber HE (1977) Die ehemalige und jetzige Brombeerflora von Mennighüffen, Kreis Herford, Ausgangsebiet der europäischen *Rubus*-Forschung durch KEA Weihe (1779–1834).

[B23] Wilhelm G Rericha L (2020) Illustrated Glossary of Botanical Terms. Flora of the Chicago Region, A Floristic and Ecological Synthesis, fourth Edition. Indiana Academy of Sciences and Conservation Research Institute, USA, 1–43. http://conservationresearchinstitute.org/forms/CRI-FLORA-Glossary.pdf [Accessed 02.08.2021]

