# Supplementary material for: ﻿Rediscovery of Rubuspendulus Rusby (Rosaceae) and a new record for the flora of Ecuador and Peru
Source: PhytoKeys. 2023 Jun 2;227:109–22. doi: 10.3897/phytokeys.227.100859 (PMC10257138; doi:10.3897/phytokeys.227.100859)
Supplement: Supplementary material 1 — Taxonomic identificacion key for Ecuadorian Rubus’ species [file phytokeys-227-109_article-100859__-s001.docx]

Supplementary Table 1. Taxonomic Key for Ecuadorian *Rubus*’ species

| 1. | Stipules linear-falcate, ovate, or suborbicular; leaves simple or 3-foliolate | **2** |
| --- | --- | --- |
| – | Stipules subulate or filiform; leaves 3-foliolate, palmately 5-foliolate, or imparipinnate | **11** |
| 2. | Leaves simple | **3** |
| – | Leaves 3-foliolate | **7** |
| 3. | Stipules linear-falcate | *R. loxensis* |
| – | Stipules asymmetrically ovate | **4** |
| 4. | Upper leaf surface bullate | *R. azuayensis* |
| – | Upper leaf surface not bullate | **5** |
| 5. | Lower leaf surface pannose-tomentose | *R. acanthophyllos* |
| – | Lower leaf surface glabrous or sparsely pilose on veins | **6** |
| 6. | Flowers solitary or rarely in inflorescences 2–3 cm long, with fewer than four flowers | *R. coriaceus* |
| – | Inflorescences 5–9 cm long, with more than five flowers | *R. laegaardii* |
| 7. | Flowers solitary or in few-flowered lax inflorescences; sepals as long as or longer than petals | **8** |
| – | Flowers in simple or compound, compact inflorescences; sepals shorter than petals | **10** |
| 8. | Stipules ovate; flowers usually solitary or sometimes in inflorescences with 2–4 flowers | *R. glabratus* |
| – | Stipules suborbicular; inflorescences with more than four flowers | **9** |
| 9. | Lower leaflet surface glabrous or sparsely pilose; unarmed sepals | *R. roseus* |
| – | Lower leaflet surface tomentose or villous; prickly sepals | *R. nubigenus* |
| 10. | Leaves and inflorescences pubescent, prickly sepals | *R. nubigenus* |
| – | Leaves and inflorescences glabrous, unarmed sepals | *R. compactus* |
| 11. | Drupelets united and falling collectively from dry receptacle | **12** |
| – | Drupelets remaining on the fleshy receptacle and falling together with it | **14** |
| 12. | Leaves 3-foliolate; fruit yellow | *R. ellipticus* |
| – | Leaves imparipinnate, 5- or 7-foliolate; fruit pinkish-purple to black or red | **13** |
| 13. | Lower leaf surface pannose with stipitate glands, whitish; stem pruinose | *R. niveus* |
| – | Lower leaf surface sparsely pilose with subsessile and sessile glands, greenish; stem not pruinose | *R. rosifolius* |
| 14. | Stems strongly glaucous (whitish); abaxial surface strongly whitish-pannose; fruits with > 70 drupelets | **15** |
| – | Stems not glaucous (whitish); abaxial surface glabrous, villous, velutinous, tomentose, pilose or greenish-pannose; fruits with < 70 drupelets (except *R. pendulus*) | **16** |
| 15. | Stems pruinose; stipules < 15 mm long; petals white; drupelets > 3 mm long | *R. glaucus* |
| – | Stems not pruinose; stipules > 20 mm long; petals fuchsia; drupelets < 3 mm long | *R. longistipularis* |
| 16. | Red, yellow or orangish, eglandular setose hairs all over the plant | **17** |
| – | Setose hairs absent all over the plant | **18** |
| 17. | Upper leaf surface bullate; leaf abaxial surface glabrous with villous-hirsute hairs only on the veins; less than 60 flowers per inflorescence; sepals mucronulate | *R. pendulus* |
| – | Upper leaf surface not bullate; leaf abaxial surface pannose; more than 60 flowers per inflorescence; sepals apiculate or acuminate | *R. urticifolius* |
| 18. | Basal leaves 3-foliolate; inflorescences few-flowered, usually fewer than 30 flowers per inflorescence (except for *R. maquipucunensis*) | **19** |
| – | Basal leaves 5-foliolate, rarely 3-foliolate; inflorescences many-flowered, usually more than 40 flowers per inflorescence | **23** |
| 19. | Stems glabrous or puberulent; leaf adaxial surface eglandular | *R. megalococcus* |
| – | Stems tomentose, velutinous, villous, pilose; leaf adaxial surface with sessile and subsessile glands | **20** |
| 20. | Leaflets with fewer than nine pairs of secondary veins; stems pilose; petals greenish-white | *R. adenothallus* |
| – | Leaflets with more than 10 pairs of secondary veins; stems tomentose, velutinous, or villous; petals reddish-violet, white, or pink | **21** |
| 21. | Vine or climbing shrub; leaflets broadly elliptic or broadly ovate to elliptic with more than 14 pairs of secondary veins (except in hanging leaves) | *R. maquipucunensis* |
| – | Scandent shrub; leaflets ovate to slightly elliptic with less than 13 pairs of secondary veins | **22** |
| 22. | Leaflet surface velutinous or tomentose, with sessile and subsessile glands | *R. bogotensis* |
| – | Leaflet surface sparsely villous or pilose, eglandular | *R. peruvianus* |
| 23. | Stems and branches glandular | **24** |
| – | Stems and branches eglandular | **26** |
| 24. | Stems densely covered with long-stipitate glands | *R. adenotrichos* |
| ­– | Stems with scattered, short-stipitate glands | **25** |
| 25. | Petiole pulvinate; base of leaflets asymmetrical | *R. killipii* |
| – | Petiole not pulvinate; base of leaflets rounded | *R. floribundus* |
| 26. | Lower leaflet surface glabrous; leaflets with 7–10 pairs of secondary veins | *R. killipii* |
| – | Lower leaflet surface pubescent; leaflets with 10–18 pairs of secondary veins | **27** |
| 27. | Leaflets with 10–12 or rarely 14 pairs of secondary veins, leaf margins serrate | *R. floribundus* |
| – | Leaflets with 14–18 pairs of secondary veins, leaf margins serrulate | *R. boliviensis* |
