# Supplementary material for: ﻿Rediscovery of Rubuspendulus Rusby (Rosaceae) and a new record for the flora of Ecuador and Peru
Source: PhytoKeys. 2023 Jun 2;227:109–22. doi: 10.3897/phytokeys.227.100859 (PMC10257138; doi:10.3897/phytokeys.227.100859)
Supplement: Supplementary material 2 — Comparison of main morphological characters between Rubuspendulus, R.urticifolius, R.porphyromallos, R.bogotensis and R.mollifrons [file phytokeys-227-109_article-100859__-s002.docx]

Supplementary Table 2. Comparison of main morphological characters between *Rubus pendulus*, *R. urticifolius*, *R. porphyromallus*, *R. bogotensis* and *R. mollifrons*. ND=No Data Available

|  | ***Rubus pendulus* Rusby** | ***Rubus urticifolius* Poir.** | ***Rubus porphyromallos* Focke** | ***Rubus bogotensis* Kunth** | ***Rubus mollifrons* Focke** |
| --- | --- | --- | --- | --- | --- |
| **Habit** | Woody vine or scandent or climbing shrub | Scrambling shrub | Shrub | Scandent shrub | Climbing shrub |
| **Stem pubescence** | Reddish setose and hirsute | Reddish setose | Reddish-villous and tomentose | Tomentose or velutinous | Tomentose |
| **Glands on branches** | Eglandular or with some setose hairs ending in a gland | Eglandular or with scattered sessile and subsessile glands | Eglandular | Short-stipitate glands covering most of the plant | ND |
| **Leaf base** | Subcordate or asymmetrically subcordate | Rounded | Emarginate or subcordate | Rounded or slightly truncate | Subcordate |
| **Bullate leaves** | Yes | No | No | No | No |
| **Leaf adaxial surface pubescence** | Sparsely villous-hirsute on each bubble, and densely villous hirsute on the veins | Surface sparsely pilose and tomentose on the veins | Surface strigose | Surface sparsely velutinous or tomentose, mostly on the veins | Surface densely grayish-pannose |
| **Leaf abaxial surface pubescence** | Surface glabrous and villous with red setose hairs only on the veins | Surface pannose and tomentose on the veins | Surface softly grayish-pannose | Surface velutinous or tomentose, mostly on the veins | Surface grayish-pannose |
| **Basal petiolule length** | 0.3–0.7 cm | 0.4–2.37 cm | 2–2.5 cm | Absent | Absent |
| **Lateral petiolule length** | 0.7–2.97 cm | 0.31–3.18(–5.12) cm | ≈ 4 cm | 0.36–1.43 cm | ≈ 1 cm |
| **Terminal petiolule length** | 2.8–5.1 cm | 1.59–4.52(–7.37) cm | 5–6 cm | 2.11–4.57 cm | ≈ 2 cm |
| **Flowers per inflorescence** | 6–56 | 60–150 | No data available | 12–15(–40) | 1 or pauciflora |
| **Petal shape** | Obovate-lanceolate to elliptic | Broadly obovate | Obovate | Spathulate to suborbicular | Elliptic |
| **Sepal pubescence** | Abaxial: villous-sericeous, and tomentose towards the apex and the margin  Adaxial: tomentose | Abaxial: tomentose-pannose  Adaxial: pannose | Grayish-tomentose, not villous | Abaxial: tomentose or pilose  Adaxial: pannose | Grayish-tomentose |
| **Sepal apex** | Mucronulate | Apiculate or acuminate | ND | Acuminate | ND |
| **Fruit size** | 7.8–15.4 × 6.6–11 mm | 7–10 × 6–9 mm | ND | 15–20 × 10–20 | ND |
| **Drupelets per fruit** | 66–115 | 30–50 | ND | 10–35 | ND |
| **Drupelet size** | 2.1–4.3 × 1.1–2.8 mm | 1.5–3 × 1–2 mm | ND | 5–8 × 4–6 mm | ND |
